# The sodium channel gene family is specifically expressed in hen uterus and associated with eggshell quality traits

**DOI:** 10.1186/1471-2156-14-90

**Published:** 2013-09-24

**Authors:** Yan-Feng Fan, Zhuo-Cheng Hou, Guo-Qiang Yi, Gui-Yun Xu, Ning Yang

**Affiliations:** 1 National Engineering Laboratory for Animal Breeding and MOA Key Laboratory of Animal Genetics and Breeding, China Agricultural University, Beijing 100193, China

**Keywords:** Sodium channel gene family, Eggshell quality, Cation transportation, Phenotypic variance, Uterus

## Abstract

**Background:**

Eggshell quality is important for the poultry industry. During eggshell formation a mass of inorganic minerals is deposited. The Sodium Channel (SCNN1) gene family plays an essential role in cation transportation. The objective of this study was to investigate the pattern of expression of members of the SCNN1 gene family, their variation and their effects on eggshell quality.

**Result:**

The highest expression of SCNN1a, SCNN1b, and SCNN1g genes were in the active uterus during eggshell mineralization, while SCNN1d showed its highest expression level in the quiescent uterus (no egg present). Nineteen candidate SNPs from the four genes were genotyped in a population of 338 White Leghorn layers. Association analysis between SNPs (haplotypes/diplotypes) and eggshell traits was performed. Among seven significant SNPs, five SNPs were associated with eggshell strength, eggshell thickness, eggshell percentage or/and egg weight, while the other two SNPs within SCNN1d were only associated with eggshell percentage. These SNPs had a 0.25-6.99% contribution to phenotypic variance, depending on the trait. In haplotype analysis, SCNN1b and SCNN1d were associated with egg weight. The SCNN1b and SCNN1g were significantly associated with eggshell weight while only SCNN1g explained 2.04% of phenotypic variance. All the alleles of the members of SCNN1 gene family were associated with eggshell percentage and eggshell thickness, and others members had an association with eggshell strength except for SCNN1a. The contribution of different haplotypes of the SCNN1 gene family to eggshell phenotypic variance ranged from 0.09% to 5.74%.

**Conclusions:**

Our study indicated that the SCNN1 gene family showed tissue expression specificity and was significantly associated with eggshell traits in chicken. This study provides evidence that genetic variation in members of the sodium channel can influence eggshell quality.

## Background

Chicken eggs have a high nutritive value as a human food source and provide a less expensive animal protein for consumers than other foodstuffs such as meat and milk [1]. Improving eggshell quality is critically important for the poultry industry. A fragile eggshell brings economic loss at each stage of the production process. It has been estimated that in the process of egg production more than ten percent of total eggs could not be collected or are lost [2]. The eggshell has been shaped through evolution to resist physical and pathogen challenges from the external environment, to satisfy the metabolic and nutritional needs of the developing embryo by regulating gas and water exchange, and to serve as a calcium store [3,4].

The eggshell is a complex and highly structured calcitic bioceramic. As the forming egg traverses the oviduct, the eggshell membranes are assembled in the isthmus and the eggshell is deposited in the uterus [3]. About 94% of eggshell mineral is calcium carbonate, with other inorganic minerals such as magnesium carbonate, calcium phosphate and magnesium phosphate [5]. Ion transportation plays a very important role in the process of eggshell formation. The ion channel superfamily includes voltage-gated K^+^ channels, voltage-gated Ca^2+^ channels, Na^+^ channels and non voltage-gated Na^+^ channels, etc. Amiloride-sensitive Na^+^ channels are a diverse group of ion channels which are essential for controlling the regulation of Na^+^ transport into cells and across epithelia [6]. In the quail uterus, net Na^+^ flux to the serosal side is observed in vitro [7]. Na^+^ was actively transported across the uteruine epithelium into the plasma while net Ca^2+^ secretion rose progressively when perfused Na^+^ concentrations were increased. These observations suggested a positive influence of Na^+^ absorption on net Ca^2+^ secretion in the avian uterus [8]. Furthermore, new evidence suggests that the concentration and transfer of Na^+^ can directly influence the transportation of calcium and bicarbonate ions in chicken uterus [9].

The amiloride-sensitive Na^+^ channels are made up of four subunits α, β, γ, and δ (encoded by the SCNN1a, SCNN1b, SCNN1g and SCNN1d genes, respectively) [10]. These four genes are all members of the Sodium Channel Gene Family (SCNN1 gene family) [11,12]. The three distinct but similar subunits α, β, and γ can form a non-voltage gated sodium channel, for example, the epithelial sodium channel (ENaC). The amiloride-sensitive ENaC is a membrane constituent of many salt-reabsorbing epithelia and its activity limits the salt-reabsorption rate [13]. The pore-forming α subunit spans the cell membrane; with the accessory β and γ subunits whose extracellular domains interact with the loops of the α subunits, they together compose a heterotrimeric functional channel [14]. The δ subunit has similar features as the α subunit.

In order to deposit eggshell, the uterus mucosa must transfer Ca^2+^ from the plasma to the uterine lumen. Previous studies illustrated that the ion concentrations (mainly including Ca^2+^, Na^+^ ,K^+^, HCO_3_^-^ and Cl^-^) of the uterine fluid change during the different stages of calcification [9]. One mechanism of Ca^2+^ transportation across the uterine mucosa is Na^+^/Ca^2+^ exchange, which increases the Ca^2+^ efflux indirectly by creating an Na^+^ gradient providing energy [14]. However, it has been reported that net Ca^2+^ secretion might not be critically dependent upon Na^+^ absorption [8]. As the ENaC is a non-voltage gated channel and a regulator of fluid volume, it transports Na^+^ ions from the lumen into the cell in order to establish a voltage difference and maintain osmolarity of either side of the cell's membrane [15]. Then it indirectly helps the Ca^2+^ transportation and secretion. The disruption of sodium reabsorption by specific inhibitors in perfused uterus or in vitro causes reduced Ca^2+^ secretion [16,17], revealing a strong relationship between Na^+^ and Ca^2+^ transfer and validating the putative presence of Na^+^/Ca^2+^ exchangers in uterine cells. Recent study showed that expression of members of the SCNN1 gene family increased significantly in the uterus compared with magnum and duodenum during the active phase of calcification. Moreover, SCNN1G was expressed at a higher level in the presence of eggshell calcification than in its absence of eggshell calcification [9].

Based on this assembly of evidence to support a role for Na^+^ in Ca^2+^ transport during eggshell formation, we hypothesized that the SCNN1 gene family could affect eggshell quality traits. In the current study, our goals were to investigate the tissue expression patterns of the SCNN1 gene family, to identify polymorphisms in each gene and to study their association with chicken eggshell quality traits.

## Results

### Expression pattern of SCNN1 gene family

**Figure 1:**
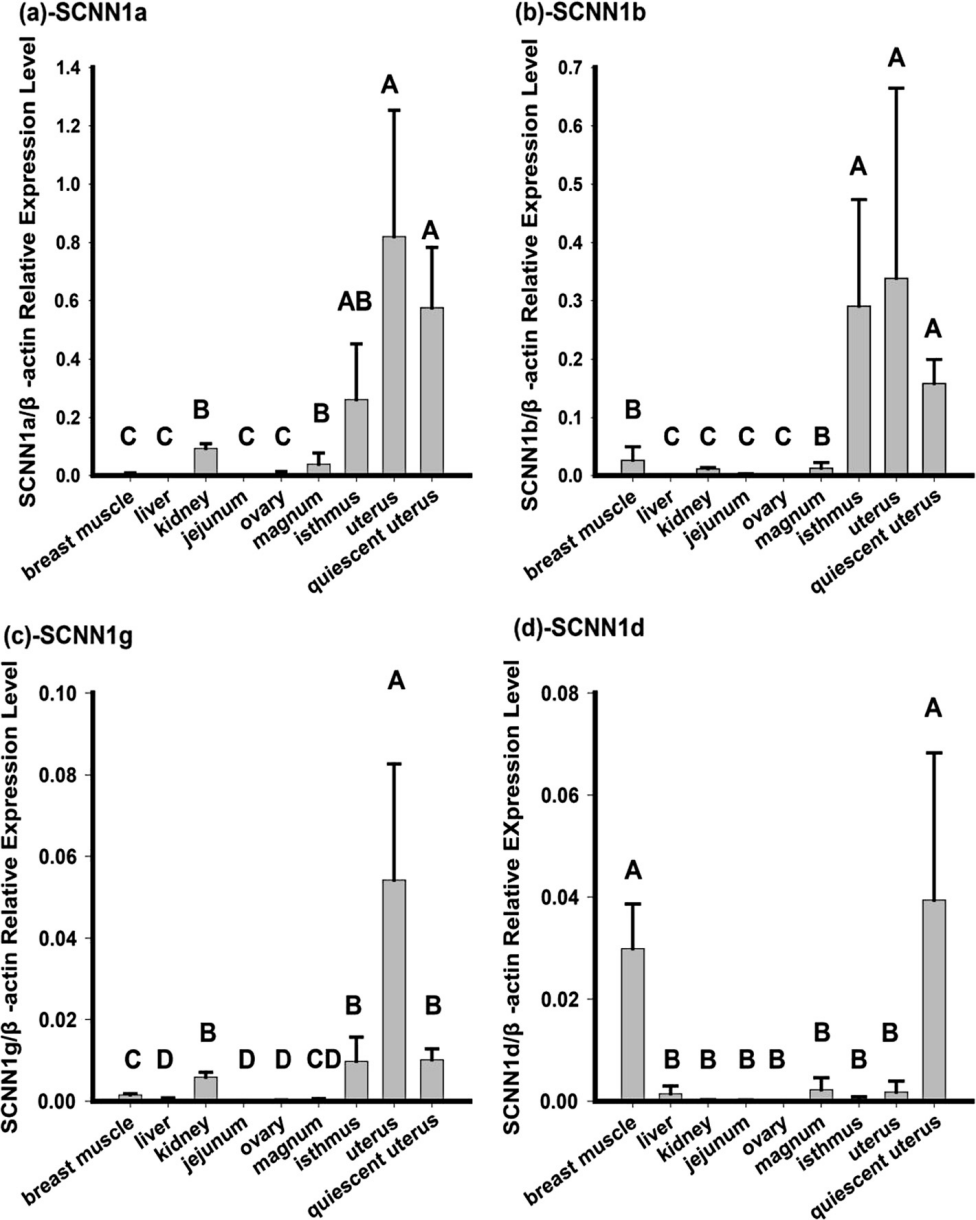
**Comparison of relative expression of SCNN1 gene family members vs β-actin measured by qPCR in different tissues.** The kidney was collected from four normal 49-Week-old White Leghorn layers while all others tissues, breast muscle, liver, jejunum, ovary, magnum, isthmus, active uterus (during eggshell deposition) and quiescent uterus (no egg present at time of tissue collection) were collected from four normal 55-week-old White Leghorn layers. The y-axis indicates the relative expression level of each SCNN1 family member. Different letters (A,B,C, D) represent significant difference (P < 0.05). Panel **(a)**, Panel **(b)**, Panel **(c)** and Panel **(d)** represents the expression of the SCNN1a, SCNN1b, SCNN1g and SCNN1d, respectively.

**Figure 2:**
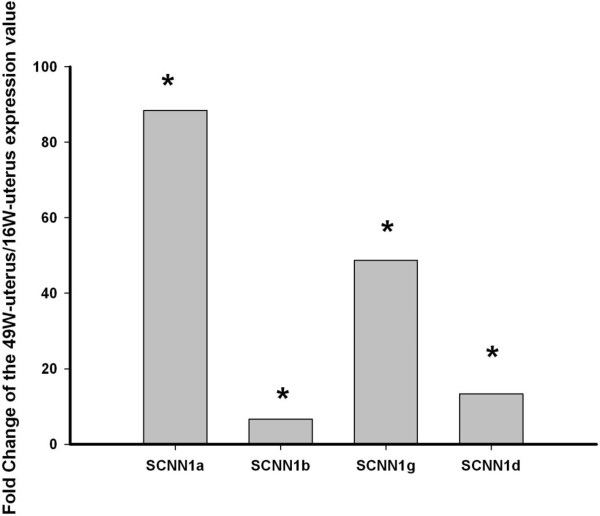
**Fold change of the relative expression by qPCR of SCNN1 gene family members in active uterus (eggshell mineralizing) of 49-week-old hens compared to 16-week-old pullets.** Expression in the 49-week-old adult hen active uterus was compared with expression in pullet uterus. * represents a significant difference (p < 0.05). The expressions of all four genes are significantly increased in 49-week-old adult hens compared to 16-week-old pullets.

The expression status of the SCNN1 gene family was analyzed by real time quantitative PCR (qPCR). As shown in Figure 1, results revealed that three family members (SCNN1a, SCNN1b and SCNN1g) had a higher expression level in active uterus than in other chicken tissues. The highest relative expression of the SCNN1g gene was detected in the active uterus during eggshell deposition (Figure 1c) (p < 0.05 for all comparisons). The highest relative expression of SCNN1a and SCNN1b were in active uterus, but they were not significantly different from the quiescent uterus (egg in magnum but not uterus) and isthmus. On the other hand, the highest relative expression of SCNN1d was observed in quiescent uterus (Figure 1d). If the SCNN1 gene family is involved in eggshell formation, it should be expressed in the uterus at a higher level in the laying stage uterus than the developing immature uterus (pullet). The uterine expression level of all SCNN1 gene family members was significantly increased in 49-week adult hens compared to 16-week pullets (Figure 2). We compared the expression level of the four different SCNN1 genes between the active and quiescent uterus during a daily laying cycle. The relative expression levels of SCNN1b, SCNN1d and SCNN1g were far less than that of SCNN1a (p < 0.05) in both active and quiescent uterus (Additional file 1: Figure S1). SCNN1b is the second most highly expressed gene among the four members in both active and quiescent uterus (Additional file 1: Figure S1). At the same time we compared the gene expression in active uterus and kidney tissues from the 49-week adult hens. The results showed that the relative expression of all members of this gene family in kidney were significantly lower than in uterus (p < 0.05) (Figure 1).

Our qPCR results illustrated that all members of the SCNN1 gene family had a relatively abundant expression in both uterus and isthmus. Analysis of their expression supported our hypothesis that these genes had a potential role in eggshell formation. Therefore we performed an association analysis between SNPs/haplotypes/diplotypes and egg weight and eggshell quality traits.

### SNP detection and association with egg weight and eggshell quality traits

Basic statistical analyses of eggshell quality traits are presented in Table 1. As there were some missing data, the sample size varied depending on the of each trait was not same as each other. Phenotypes of the samples were in the normal range.

**Table 1:** Descriptive statistics for chicken egg weight and eggshell traits

**Trait**^**1**^	**Sample number**	**Mean**	**SD**^**2**^	**Minimum**	**Maximum**
EW (g)	319	55.68	3.81	44.30	68.05
ESW (g)	294	7.47	0.76	4.60	9.89
ESP (%)	294	13.96	1.27	9.83	18.54
ESS (Kg/cm^3^)	314	3.07	0.62	1.28	4.65
EST (mm)	313	0.32	0.02	0.25	0.37

^1^EW = Egg Weight; ESW = Eggshell Weight; ESP = Eggshell Percentage; ESS = Eggshell Strength.

EST = Eggshell Thickness; % indicates these traits relative to EW.

^2^SD = standard deviation.

Of the 19 SNPs genotyped, SNP rs15731913 was not detected, SNP rs15731904 had no polymorphism (allele G frequency = 1), rs14282986 and rs14075352 were only detected in a few individuals in the studied population. Single-marker association analysis revealed that in the overall cohort, seven SNPs were significantly associated (p < 0.05) with at least one of the eggshell quality traits (Table 2). The CPV of these significantly associated SNPs ranged from 0.25% (rs13532838 to eggshell thickness (EST)) to 6.99% (rs14282978 to eggshell percentage (ESP)). As shown in Table 2, rs13532836 (SCNN1b), rs14282978 (SCNN1d), rs15181931 (SCNN1d), and rs15181934 (SCNN1d) were significantly associated with ESP. Of the four SNPs, only rs13532836 and rs15181931 were significantly associated with egg weight (EW) (the contributions to phenotypic variance (CPV) were 1.04% and 0.83% respectively).

**Table 2:** Means for eggshell-quality traits among genotypes and the SNP contribution to phenotypic variance (CPV) of the single-nucleotide polymorphism

**Gene**	**SNP**	**Trait**^**1**^	**Genotype(number)/Mean(SD)**^**2**^			**CPV**^**3 **^**(%)**
SCNN1b	rs13532836		CC(214)	TC(69)	TT(53)	
		ESP	13.95(1.30)^ab^	13.81(1.35)^b^	14.05(1.18)^a^	NA
		EW	55.58(3.88)^ab^	56.53(4.03)^b^	55.19(3.69)^a^	1.04
SCNN1b	rs13532838		CC(81)	TC(164)	TT(90)	
		ESP	13.84(1.45)^b^	14.08(1.30)^a^	13.89(1.04)^ab^	0. 28
		ESS	2.96(0.60)^b^	3.07(0.59)^ab^	3.16(0.68)^a^	1.14
		EST	0.31(0.02)^b^	0.32(0.02)^ab^	0.32(0.02)^a^	0.25
SCNN1b	rs14075350	EST	CC(151)	CT(158)	TT(28)	0.60
			0.32(0.02)^a^	0.31(0.02)^b^	0.32(0.02)^ab^	
SCNN1g	rs15009191		CC(232)	CT(91)	TT(13)	
		ESP	13.93(1.20)^ab^	14.09(1.47)^a^	13.65(1.16)^b^	NA
		EST	0.32(0.02)^a^	0.31(0.02)^b^	0.31(0.02)^b^	3.24
SCNN1d	rs15181931		CC(179)	CA(126)	AA(31)	
		ESP	14.16(1.34)^a^	13.81(1.22)^b^	13.52(1.00)^c^	3.96
		EW	55.36(3.91)^a^	55.78(3.74)^ab^	56.80(3.42)^b^	0.83
SCNN1d	rs14282978		CC(1)	CT(33)	TT(302)	
		ESP	13.99(0.00)^ab^	13.44(1.08)^b^	14.02(1.28)^a^	6.99
SCNN1d	rs15181934		CC(135)	TC(19)	TT(183)	
		ESP	13.83 (1.16)^b^	14.16(1.34)^c^	13.33(1.15)^a^	6.35

^1^EW = Egg Weight; ESP = Eggshell Percentage; ESS = Eggshell Strength.

EST = Eggshell Thickness after correction; % indicates these traits relative to Egg Weight.

^2^Different superscript (a, b, c) in the same row means phenotypes are significantly different between different genotypes (P < 0.05).

^3^CPV = SNP contribution to phenotypic variance (%).

Interestingly, among the seven SNPs which were significantly associated with egg weight and eggshell quality traits, we found that the SNPs associated with eggshell strength (ESS) or EST were all located in SCNN1b or SCNN1g genes. SNP rs13532838 of SCNN1b revealed an association with ESS and EST (P = 0.011 and 0.033, respectively). SNP rs14075350 of SCNN1b had an association with EST (P = 0.045), and SNP rs15009191 of SCNN1g was significantly associated with EST (P = 0.008). We also observed an overdominance effect of the heterozygote of SCNN1d (rs15181934) on eggshell percentage.

Multiple-markers in each gene association analysis were performed to promote our understanding of each gene’s effect on egg weight and eggshell quality traits. Additional file 2: Table S1 shows the major haplotypes (frequency > 1%) and genotypes (frequency > 0.5%) in all four genes. Because each gene contained different numbers of SNPs which were in LD (Additional file 3: Figure S2), the haplotype numbers of each gene varied. In each gene, there was a main haplotype (frequency > 30%). The results indicated that there were significant associations between haplotype and eggshell quality traits. As there were so many and complicated comparison results, we only report the comparisons between the most significantly different diplotypes (Table 3). Almost all haplotypes in our study were not in Hardy-Weinberg equilibrium. Possible reasons are discussed later. The SCNN1b (p < 0.05) and SCNN1d (p < 0.01) genes had an association with EW and SCNN1d had a 0.62% CPV on EW. The SCNN1b (p < 0.05) and SCNN1g (p < 0.01) genes had an association with ESW, ESP, ESS and EST traits and both of them had a main CPV (1.64%, 5.43%, respectively) on trait ESS. On the EST trait, compared with the CPV of SCNN1b (0.78%), the gene SCNN1g had a larger CPV (0.82%). The SCNN1a gene (p < 0.01) was significantly associated with ESP and EST traits. The SCNN1d (p < 0.05) gene showed a significant association with ESP, ESS, and EST traits while it only had a major CPV (5.74%) on ESP trait.

**Table 3:** Means for eggshell-quality traits among genotypes and the contribution to phenotypic variance (CPV) of the diplotypes

**Trait**^**1**^	**Gene**	**Diplotypes(number)**	**Mean(SD)**^**2**^	**P-value**	**CPV**^**3 **^**(%)**
EW	SCNN1b	H2H6(15)	56.48(3.06)^a^	0.0200	NA
		H8H8(21)	53.88(2.88)^b^		
	SCNN1d	H2H5(8)	58.96(3.39)^a^	0.0024	0.62
		H4H4(174)	55.36(3.86)^b^		
ESW	SCNN1b	H7H4(6)	7.84(0.39)^a^	0.0148	NA
		H6H6(5)	6.85(0.48)^b^		
	SCNN1g	H5H7(14)	7.84(1.03)^a^	<0.0001	2.04
		H11H1(5)	6.19(1.15)^b^		
ESP	SCNN1a	H1H2(8)	14.53 (1.64)^a^	0.0025	NA
		H2H4(30)	13.68(1.11)^b^		
	SCNN1b	H3H4(6)	13.67(0.89)^a^	0.0006	0.09
		H1H6(8)	13.54(0.87)^b^		
	SCNN1d	H4H4(174)	14.17(1.34)^a^	<0.0001	5.74
		H1H5(6)	12.76 (0.93)^b^		
	SCNN1g	H2H2(49)	13.71 (0.94)^a^	<0.0001	NA
		H11H1(5)	13.36(0.34)^b^		
ESS	SCNN1b	H3H4(6)	3.18(0.65)^a^	0.0021	1.64
		H6H6(5)	2.60(0.30)^b^		
	SCNN1d	H3H5(99)	3.11(0.05)^a^	0.0278	NA
		H5H5(23)	2.85(0.11)^b^		
	SCNN1g	H1H9(9)	3.64(0.73)^a^	<0.0001	5.43
		H2H6(12)	2.61(0.50)^b^		
EST	SCNN1a	H1H2(8)	0.33 (0.02)^a^	0.0085	NA
		H4H4(165)	0.32 (0.02)^b^		
	SCNN1b	H3H4(6)	0.32(0.03)^a^	0.0002	0.78
		H5H1(12)	0.30(0.03)^b^		
	SCNN1d	H1H5 (6)	0.30 (0.03)^a^	0.0069	NA
		H4H4(174)	0.32(0.02)^b^		
	SCNN1g	H1H9(9)	0.33(0.02)^a^	0.0021	0.82
		H5H5(13)	0.31 (0.02)^b^		

^1^EW = Egg Weight; ESW = Eggshell Weight; ESP = Eggshell Percentage after correction; ESS = Eggshell Strength; EST = Eggshell Thickness after correction; % indicates these traits relative to Egg Weight.

^2^Different superscript (a, b) means significantly different between different diplotypes of the same gene for certain trait.

^3^CPV = SNP contribution to phenotypic variance (%).

## Discussion and conclusions

Some efforts were performed to pursue the markers which are related with eggshell qualities. Polymorphisms of the eggshell organic matrix genes were considered to be related with eggshell breaking strength, eggshell thickness, dynamic stiffness [18]. A genome-wide SNP scan found some novel loci affected the eggshell quality [19]. These investigations are helpful to identify loci which are potentially useful in the breeding. However, current studies are very limited in terms of the population and studied genes. This study focused on the SCNN1 gene and their effects on the eggshell quality. In general, it is observed that the egg production rate is reduced with increasing hen age, and the incidence of thin-shelled and cracked eggs is markedly increased [20]. However, the plasma levels of ionized Ca^2+^ do not significantly change in hens between 33 and 122 weeks of age. Thus, the decreased eggshell thickness may involve changes in shell gland function rather than availability of Ca^2+^ for secretion [21]. A declining ability of the epithelium to transport Ca^2+^ might be one of the major reasons for decreasing eggshell quality in older hens. Could non-Ca^2+^ transporting genes related to ion transport affect the final eggshell quality? This pilot study on sodium channel related genes provides evidence to address this question.

Previous studies showed that SCNN1a, SCNN1b and SCNN1g are highly expressed in the uterus during the eggshell formation [9,22] while SCNN1g expression increases quickly during the eggshell formation stage. Genes highly and specifically expressed in a given tissue likely contribute to its development and function [15]. Our qPCR results indicated that the genes encoding the ENaC subunits, SCNN1a, SCNN1b, SCNN1g and SCNN1d, are all highly expressed in uterus (Figure 1). A strong relationship between uterine Na^+^ and Ca^2+^ transfers has been previously established [16,17], and the Na^+^/Ca^2+^ exchangers are likely present in uterine cells [9]. Na^+^ was actively transported across the uterus epithelium into the plasma while net Ca^2+^ secretion rose progressively when perfused Na^+^ concentrations were increased [8]. The Na^+^/K^+^ ATPase responsible for Na^+^ re-absorption in the plasma membrane has been characterized and is upregulated during the period of shell calcification [14]. In this study we found that expression of all members of the SCNN1 gene family also increased significantly during eggshell formation in the uterus (Figure 2). The rates of Na^+^ and anion transport are altered with age and in response to molting, which could potentially contribute to the decrease in shell thickness often associated with eggs from older birds [21]. These observations suggested a positive influence of Na^+^ absorption on net Ca^2+^ secretion [8]. Expression results and Na^+^ changes during the laying period strongly suggests that the SCNN1 gene family is involved in eggshell formation via influencing Ca^2+^ transport.

This study also explored association of different genotypes of the members of the SCNN1 gene family with eggshell qualities. We searched the AnimalQTL database [23] to find the potential QTLs which are closely related with SCNN1 gene regions. However, we didn't find the SCNN1 gene fall in any known QTL regions and also are not closely related QTLs in the current researches. Our association analysis results between SNPs/haplotypes and eggshell traits demonstrated that the SCNN1a, SCNN1b and SCNN1g mainly had effects on eggshell thickness and eggshell strength while the SCNN1d gene mainly had an effect on eggshell percentage. One SNP (SCNN1d, rs15181934) has overdominance effects on eggshell percentage. This effect might be useful for commercial breeding, especially for designing line crosses. When we constructed diplotypes based on the haplotypes, we found many different diplotypes. Our association analysis based on these diplotypes might be affected by the relatively small number of observations for some diplotypes.

However, almost all the SNPs were not in Hardy-Weinberg equilibrium in the population we studied. As our breeding population has suffered artificial selection, this is similar to most modern poultry breeding populations. Moreover, breeding populations generally are nonrandomly mated, with a relatively small population (around 800 birds per generation). All these reasons could cause some genes to not be in Hardy-Weinberg equilibrium [24]. Could the lack of HWE be caused by genotyping errors? We added replications for all SNPs of 4 randomly selected individuals to validate the accuracy of the genotyping methods used in this study. The genotyping method used in this study has been shown to be accurate in many other studies of this field [25-27]. Furthermore, we also used the direct sequencing method to verify the genotype accuracy for two SNPs (rs15181934, n=128; rs14075350, n=64) in randomly selected individuals. Error rate ranged from 7.8% (rs14075350)-11.2% (rs15181934) for two randomly selected SNPs. The error rate used in this study is comparable with other studies which used the same method. We manually checked the distribution of the genotyping error for two SNPs, and found the genotype errors generally are consistent with the genotype distribution in the whole samples. This means the genotyping errors didn’t show any bias trends for different genotypes. These randomly happened errors didn’t change the final conclusions. It is likely that the SCNN1 gene family has undergone selection related to breeding programs to improve eggshell quality traits.

In summary, members of the SCNN1 gene family are highly expressed in the adult hen active uterus compared to the immature pullet uterus. SCNN1a, SCNN1b and SCNN1g were more highly expressed in the active hen uterus than any other tissue in this study. We identified 7 SNPs that could influence eggshell quality and/or egg weight in the White Leghorn population. The results of our expression and association study provide evidence to suggest that this Sodium Channel can affect eggshell quality, especially eggshell strength and eggshell thickness.

## Method

### Animal sampling and data collection

Oviposition time was recorded daily in one week for 20 random selected 55-week-old White Leghorn layers from a relatively large population (around 500 chickens). In order to observe gene expression changes during uterus development, we also randomly selected four 16-week-old pullets (sexually immature, non-egg laying) and four 49-week-old White Leghorn layers (mature, egg producing) to obtain uterus samples. Kidney tissues were collected from four 49-week-old hens. Liver, breast muscle, jejunum, ovary, magnum, isthmus and uterus (containing an egg in the process of eggshell mineralization-active uterus) were collected from four 55-week-old hens 18–20 hours post ovulation, to represent overall gene expression levels. Another four 55-week-old hens were processed 2–4 hours post ovulation to obtain four uterine samples with egg in magnum and not in the uterus (quiescent uterus). These hens were from one hatch and were reared in the same environment at the China National Center for Poultry Performance Testing. Tissue samples for expression analysis were snap-frozen in liquid nitrogen and then stored at −80°C until RNA extraction was performed. The entire sampling process from chicken sacrifice to tissue snap freezing was finished within less than 10 minutes. Total RNA from tissues was extracted with E.Z.N.ATM Total RNA Kit (OMEGA Bio-tek Inc., USA), and dissolved in diethyl pyrocarbonate (DEPC)-treated water.

Three-hundred and thirty-eight White Leghorn layers including 40 sire families, from one hatch and reared in the same environment, were utilized for association analysis. Blood samples were collected from the wing vein, quickly homogenized with the anticoagulant agent acid citrate dextrose (ACD) and then stored at −20°C. The chicken genomic DNA was extracted using a phenol/chloroform method. Birds were individually caged. At the age of 40 weeks, eggs were collected in 3 consecutive days from each hen for the measurement of egg quality. Egg weight (EW), eggshell weight (ESW), eggshell strength (ESS) and eggshell thickness (EST) were measured within 12 h after collecting eggs, as described in our previous publications [28,29], except for eggshell weight measurement. Eggshell weight refers to the wet eggshell which still contained the eggshell membranes with adhering water and albumen. Dividing EW by ESW, we get the eggshell percentage (ESP). As the ESP and EST data did not satisfy normal distribution, they were calibrated by the box-cox method when conducting T-Test [30,31].

The animal experiments were approved by the Animal Welfare Committee of China Agricultural University.

### Cloning and expression of SCNN1 gene family

cDNA was obtained by the reverse transcription polymerase chain reaction (RT-PCR) that was performed in a total volume of 50 μL, using 1 μg of total RNA, 2 μL oligo T18 primer (10 μM) (synthesized by Sangon Co. Ltd. Beijing, China), 1 μL murine leukemia virus (MLV) reverse transcriptase, 10 μL of 5 × Buffer (ZePing Bioscience & Technologies Co. Ltd., Beijing, China), 1 μL RNAase inhibitor (TaKaRa Co. Ltd., Beijing, China), 24 nmol dNTP mixture (DingGuo ChangSheng Biotech Co. Ltd., Beijing, China), and sterile deionized water. A negative control was performed with sterile water as the template in each reaction. The optimum reverse transcription thermal cycling parameters were as follows: 10 min at 25°C, 1 hour at 37°C, 5 min at 95°C in a Mastercycler gradient (Eppendorf Limited, Hamburg, Germany). The quality of cDNA was assured through the agarose gel figure and the housekeeping gene β-actin in order to avoid the genome DNA pollution. We did PCR to amplify our target gene, ligated the purified PCR products using pMD19T vector (Takara Biotechnology Co., Ltd) at 16°C for 6 hours. Then we transformed the ligated product into E.coli to get our cloning gene using the plate paint isolation methods and blue-white selection. All selected bacterial colonies are identified by PCR using the bacteria liquid as template and sequencing (Sangon Co. Ltd. Beijing, China).

All primers (Table 4) were designed using Primer Express 3.0 software (Applied Biosystem AB Inc., USA) based on the chicken full-length mRNA sequences predicted by EnsEMBL(v.62) and synthesized by Shanghai Sangon Biological Engineering Technology & Services Co. Ltd. (Shanghai, China). Each pair primer had only one amplification product. All amplification products were sequenced to confirm validity of the primers. The amplification product of every gene by our designed primers is unique in this SCNN1 gene family. PCR was performed in a total volume of 20 μL, including 1 μL of first-strand cDNA, 0.5 μL dNTP mixture(10 mM), 2 μL of 10×Taq Buffer, 0.5 μL of Taq enzyme (ZeXing Biotech Co. Ltd., Beijing, China), 0.5 μL of each primer (10 μM), and 15 μL of sterile deionized water. The optimum thermal cycling parameters were as follows: 5 min at 95°C, followed by 32 cycles of 30 s at 95°C, 30 s at 60°C, and 30 s at 72°C, and a final extension of 5 min at 72°C in a Mastercycler gradient.

**Table 4:** Primer sequences used in qPCR for SCNN1 gene family members and internal control gene β-actin

**Primer name**	**Sequence (5’→3’)**	**Tm(°C)**	**Product size (bp)**	**Position**
SCNN1a-F	TCATGTTCAGCGCCATCCT	60	175	Exon3+4 628-802
SCNN1a-R	TTCCCGCACTGCATCCA	60		
SCNN1b-F	GAAGTTCCCAGCAGTCACAGTCT	60	168	Exon2+3 277-444
SCNN1b-R	GTGGCGTTGCTGTTGTTCAG	60		
SCNN1d-F	CACCATCCACGGCACCAT	60	183	Exon1 90-272
SCNN1d-R	AACATCTTGGGCTCCGAATG	60		
SCNN1g-F	TGGGACAAAGGACAGAAAATC	60	141	Exon10+12 1450-1590
SCNN1g-R	GCCGAAGTTGGACAGAAGGA	60		
β-actin-F	TATGTGCAAGGCCGGTTTC	60	110	Exon1+2 113-222
β-actin-R	TGTCTTTCTGGCCCATACCAA	60		

The real-time quantitative PCR (qPCR) was run using the ABI 7300 system (Applied Biosystems). First-strand cDNA from RT-PCR (above) was used as template. The housekeeping gene β-actin (GenBank Reference Sequence: NC_006101.3) of chicken was used as an internal control. Each reaction mixture consisted of a total volume of 15μL with 7.5 μL of Power SYBR® Green PCR Master Mix (Applied Biosystems), 0.2 μL of each primer (10 μM), 1.5 μL of cDNA, and 5.6 μL of ultrapure ribonuclease-free water. The qPCR procedure was at 95°C for 10 min; 40 cycles of 95°C for 15 s and 60°C for 1 min; 95°C for 15 s; 60°C for 30 s; and 95°C for 15 s. Each individual sample and no-template controls were run in triplicates.

The quantitative values of each target gene were obtained from the threshold cycle (Ct). The expression values were calculated by the formula 2^−ΔΔCt^. They were normalized using the expression values for the β-actin gene to obtain the relative expression of target genes. The relative gene expression was analyzed by the double standard curves method [28,29,32]. All the expression values were transformed using log2 when conducting T-Test. Statistical analysis of differential expression between tissues was performed using the T-Test implemented in the SAS System release 8.0 (SAS Institute Inc., USA).

### Single nucleotide polymorphisms detection

We used the NCBI SNP bank (NCBI, http://www.ncbi.nlm.nih.gov/), Ensembl Data mining tool BioMart (http://www.ensembl.org/) and the UCSC Genome Bioinformatics (http://genome.ucsc.edu/ ) to conduct online searching for potential SNPs in the SCNN1 gene family DNA sequences. All SNPs had an rs# number which information could be accessed on dbSNP. All the SNP positions were reported based on the reference chicken genome (Genome assembly: WASHUC2). A high-throughput genotyping method, matrix-assisted laser desorption-ionization time-of-flight mass spectrometry (MALDI-TOF MS), was used to distinguish these SNPs genotypes. The genotypes were analyzed by MALDI-TOF MS based on the Sequenom’s MassARRAY iPLEX Platform (Sequenom, San Diego, CA). In these chip analyses, we randomly designed 4 repeats to ensure the reliability of the technology. SNPs with a genotype call rate < 85% and minor allele frequency (MAF) < 1% across all individuals were discarded. Firstly, we designed a preliminary experiment to test the polymorphism and compatibility of all SNPs in the four genes for small samples (n=50). Finally based on the preliminary experiment results (SNP frequency, position and mutation type), we identified 19 SNPs in the four genes that were used in our study (Table 5).

**Table 5:** Details of the SCNN1 gene family members and SNPs selected

**Gene**	**Gene location**	**No. of SNPs**^**1**^	**SNP rs#.**	**SNP location (Chr: bp)**	**Mutations type**	**Major allele (Fre)**^**2**^
SCNN1a	chr1: 80,034,908-80,045,394	2	rs14845039	1:80035441	Synonymous coding (C/T)	T(0.71)
			rs13886292	1:80039016	Intronic (G/C)	G(0.82)
SCNN1b	chr14: 7,002,221-7,011,192	6	rs13532836	14:7005977	Intronic (A/G)	C(0.74)
			rs13532838	14:7007309	Synonymous coding (A/G)	T(0.51)
			rs14075350	14:7011699	5’UTR (C/T)	C(0.68)
			rs13532842	14:7011842	Intronic (A/G)	A(0.64)
			rs15731904	14:7003218	Intronic (A/G)	G(1.00)
			rs15731913	14:7006347	Intronic (C/G)	NA
SCNN1d	chr21: 2,435,983- 2,440,820	4	rs14282978	21:2436106	Synonymous coding (C/T)	T(0.95)
			rs15181931	21:2438805	Intronic (T/G)	C(0.72)
			rs15181934	21:2442772	5’UTR (G/A)	T(0.57)
			rs14282986	21:2437824	Intronic (A/G)	T(0.70)
SCNN1g	chr14: 7,019,365- 7,028,441	7	rs10730783	14:7018929	3’UTR (A/G)	G(0.82)
			rs15009191	14:7019233	3’UTR (A/G)	C(0.83)
			rs15009198	14:7022273	Intronic (A/G)	C(0.77)
			rs15009204	14:7023020	Intronic (A/C)	T(0.57)
			rs15009207	14:7027589	Intronic (A/G)	T(0.56)
			rs15009209	14:7027788	Intronic (A/G)	T(0.57)
			rs14075352	14:7019820	Intronic (C/G)	C(0.90)

^1^No. of SNPs = the number of SNPs selected in each gene. All SNP positions were reported based on the reference chicken genome (Genome assembly: WASHUC2).

^2^Major allele and its frequency; NA means that the allele was not detected.

### Statistical analysis

The Hardy-Weinberg equilibrium test and frequencies of SNPs and genotypes were analyzed using the FREQ procedure of SAS 8.0 (SAS Institute Inc., Cary, NC). For the SNPs in one gene, genotype data was used in the haplotype analysis using Simwalk2 (2.91) [33,34]. The following model was designed for the association analysis between the SNP/haplotype/diplotype and eggshell quality traits using the VARCOMP procedure: *Y*_*ij*_ = *μ* + *S*_*i*_ + *G*_*j*_ + *e*_*ij*_, where Y_*ij*_ was the observed value of eggshell quality traits of the *i*^th^ individual with *j*^th^ genotype; μ was the population’s mean; S_*i*_ was the fixed effects of the *i*^th^ sire family, G_j_ was the random effects for the *j*^th^ genotype, and e_*ij*_ represented the residuals. The contribution of the SNP/haplotype/diplotype to the phenotype variation (CPV) was estimated using the equation: *CPV* = *V*_*SNP*/*haplotype*/*diplotype*_ ÷ *V*_*phenotype*_; where V_SNP/haplotype/diplotype_ and V_Phenotype_ were the SNP/diplotype and phenotypic variance, respectively. The V_Phenotype_ was calculated using the quotation: $V_{\mathrm{phenotype}}=V_{\mathrm{SNP}/\mathrm{haplotype}/\mathrm{diplotype}}+V_{\mathrm{environment}\mathrm{var}\mathrm{iances}}$.

We used the VARCOMP procedure implemented in the SAS system and the residual maximum likelihood (REML) method to estimate the variance component for each variable used in the model.

The HaploView program [35] was used in the linkage disequilibrium (LD) analysis between SNPs in same gene. The LD block was defined according to the confidence interval method [36]. The genealogy and LD results were used in statistical analysis of haplotype.

## Competing interests

The authors declare that they have no competing interests.

## Authors’ contributions

Conceived and designed the experiments: ZCH, NY, YFF. Performed the experiments: YFF. Analyzed the data: ZCH, GQY, YFF. Contributed reagents/materials/analysis tools: GYX, GQY, ZCH, NY. Wrote the paper: YFF, ZHC, NY. All authors read and approved the final manuscript.

## Supplementary Material

Additional file 1: Figure S1 Comparison of relative expression of SCNN1 gene family members vs β-actin in uterine tissues by qPCR. Gene expression compared between active uterus (during eggshell deposition) and quiescent uterus (no egg present), in tissues collected from four normal 55-week-old White Leghorn layers. The y-axis indicates the relative expression level of SCNN1 family members compared with β-actin. Vertical bars represent the mean ± SD (n = 4). # represent the a, b, d or g. The expression of SCNN1a, SCNN1b and SCNN1g were reduced to some degree in the quiescent uterus, that of SCNN1d increased about 4-fold. The relative expression levels of SCNN1b, SCNN1d and SCNN1g were far less than that of SCNN1a (P<0.05). Click here for file

Additional file 2: Table S1 Major haplotypes (frequency >1%) and diplotypes (frequency >0.5%) for SCNN1 family members. Click here for file

Additional file 3: Figure S2 Haploview plot illustrates the linkage disequilibrium of SCNN1 gene family members for the main informative SNPs. The downward-pointing Triangle black box represents LD block. The number in the square represents the value of D’. The darker the square color shows and the larger the D’ value, the higher the level of two sites linkage disequilibrium displays. All SNPs r^2^ ≥ 0.933 and their minor allele frequencies were > 0.05. In the SNP name, the first letter a, b, d and g are the abbreviation of gene SCNN1a, SCNN1b, SCNN1d and SCNN1g respectively and rs## represents the last two number of the SNP ID. Click here for file

## References

[B1] Nys Y Bain M Immerseel FV Improving the safety and quality of eggs and egg products 2011 Oxford: Woodhead Pub

[B2] Takahashi H Yang D Sasaki O Furukawa T Nirasawa K Mapping of quantitative trait loci affecting eggshell quality on chromosome 9 in an F2 intercross between two chicken lines divergently selected for eggshell strength Anim Genet 2009 40 5 779 782 10.1111/j.1365-2052.2009.01914.x 19780721

[B3] Hincke MT Nys Y Gautron J Mann K Rodriguez-Navarro AB McKee MD The eggshell: structure, composition and mineralization Front Biosci 2012 17 1266 1280 10.2741/3985 22201802

[B4] Romanoff AL Study of the Physical Properties of the Hen's Eggshell in Relation to the Function of Shell-Secretory Glands Biol Bull 1929 56 5 351 356 10.2307/1537075

[B5] Nys Y Gautron J Garcia-Ruiz JM Hincke MT Avian eggshell mineralization: biochemical and functional characterization of matrix proteins Comptes Rendus Palevol 2004 3 6–7 549 562

[B6] Benos DJ Stanton BA Functional domains within the degenerin/epithelial sodium channel (Deg/ENaC) superfamily of ion channels J Physiol 1999 520 3 631 644 10.1111/j.1469-7793.1999.00631.x 10545131 PMC2269617

[B7] Pearson T Goldner A Calcium transport across avian uterus. I. Effects of electrolyte substitution Am J Physiol -- Legacy Content 1973 225 6 1508 1512 10.1152/ajplegacy.1973.225.6.1508 4760468

[B8] Eastin WC Spaziani E On the mechanism of calcium secretion in the avian shell gland (Uterus) Biol Reprod 1978 19 3 505 518 10.1095/biolreprod19.3.505 719101

[B9] Jonchère V Brionne A Gautron J Nys Y Identification of uterine ion transporters for mineralisation precursors of the avian eggshell BMC Physiol 2012 12 1 1 17 10.1186/1472-6793-12-1 22943410 PMC3582589

[B10] Canessa CM Schild L Buell G Thorens B Gautschi I Horisberger J-D Rossier BC Amiloride-sensitive epithelial Na+ channel is made of three homologous subunits Nature 1994 367 6462 463 467 10.1038/367463a0 8107805

[B11] Waldmann R Champigny G Bassilana F Voilley N Lazdunski M Molecular cloning and functional expression of a novel amiloride-sensitive Na+ channel J Biol Chem 1995 270 46 27411 27414 10.1074/jbc.270.46.27411 7499195

[B12] Yamamura H Ugawa S Ueda T Nagao M Shimada S A novel spliced variant of the epithelial Na+ channel δ-subunit in the human brain Biochem Biophys Res Commun 2006 349 1 317 321 10.1016/j.bbrc.2006.08.043 16930535

[B13] Hummler E Beermann F Scnn1 sodium channel gene family in genetically engineered mice J Am Soc Nephrol 2000 11 suppl 2 129 134 11065344

[B14] Lavelin I Meiri N Genina O Alexiev R Pines M Na+−K+−ATPase gene expression in the avian eggshell gland: distinct regulation in different cell types Am J Physiol Regul Integr Comp Physiol 2001 281 4 R1169 R1176 11557625 10.1152/ajpregu.2001.281.4.R1169

[B15] Enuka Y Hanukoglu I Edelheit O Vaknine H Hanukoglu A Epithelial sodium channels (ENaC) are uniformly distributed on motile cilia in the oviduct and the respiratory airways Histochem Cell Biol 2012 137 3 339 353 10.1007/s00418-011-0904-1 22207244

[B16] Pearson T Goldner A Calcium transport across avian uterus. II. Effects of inhibitors and nitrogen Am J Physiol - Cell Physiol 1974 227 2 465 468 10.1152/ajplegacy.1974.227.2.465 4850851

[B17] Eastin WC Spaziani E On the control of calcium secretion in the avian shell gland (Uterus) Biol Reprod 1978 19 3 493 504 10.1095/biolreprod19.3.493 719100

[B18] Dunn IC Joseph NT Bain M Edmond A Wilson PW Milona P Nys Y Gautron J Schmutz M Preisinger R Polymorphisms in eggshell organic matrix genes are associated with eggshell quality measurements in pedigree Rhode Island Red hens Anim Genet 2009 40 1 110 114 10.1111/j.1365-2052.2008.01794.x 18828860

[B19] Liu W Li D Liu J Chen S Qu L Zheng J Xu G Yang N A genome-wide snp scan reveals novel loci for egg production and quality traits in white leghorn and brown-egg dwarf layers PLoS One 2011 6 12 e28600 10.1371/journal.pone.0028600 22174844 PMC3234275

[B20] Joyner CJ Peddie MJ Taylor TG The effect of age on egg production in the domestic hen Gen Comp Endocrinol 1987 65 3 331 336 10.1016/0016-6480(87)90117-1 3557097

[B21] Vetter AE O'Grady SM Sodium and anion transport across the avian uterine (shell gland) epithelium J Exp Biol 2005 208 3 479 486 10.1242/jeb.01409 15671336

[B22] Jonchere V Rehault-Godbert S Hennequet-Antier C Cabau C Sibut V Cogburn L Nys Y Gautron J Gene expression profiling to identify eggshell proteins involved in physical defense of the chicken egg BMC Genomics 2010 11 1 57 10.1186/1471-2164-11-57 20092629 PMC2827412

[B23] Hu Z-L Park CA Wu X-L Reecy JM Animal QTLdb: an improved database tool for livestock animal QTL/association data dissemination in the post-genome era Nucleic Acids Res 2013 41 D1 D871 D879 10.1093/nar/gks1150 23180796 PMC3531174

[B24] Hartl DL Clark AG Principles of population genetics 2007 4 Sunderland: Mass.: Sinauer Associates

[B25] Werner M Sych M Herbon N Illig T König IR Wjst M Large-scale determination of SNP allele frequencies in DNA pools using MALDI-TOF mass spectrometry Hum Mutat 2002 20 1 57 64 10.1002/humu.10094 12112658

[B26] Lu F Qian Y Li H Dong M Lin Y Du J Lin Y Chen J Shen C Jin G Genetic variants on chromosome 6p21.1 and 6p22.3 are associated with type 2 diabetes risk: a case–control study in Han Chinese J Hum Genet 2012 57 5 320 325 10.1038/jhg.2012.25 22437209

[B27] Kote-Jarai Z Saunders EJ Leongamornlert DA Tymrakiewicz M Dadaev T Jugurnauth-Little S Ross-Adams H Al Olama AA Benlloch S Halim S Fine-mapping identifies multiple prostate cancer risk loci at 5p15, one of which associates with TERT expression Hum Mol Genet 2013 22 12 2520 2528 10.1093/hmg/ddt086 23535824 PMC3658165

[B28] Zhang Y Hou ZC Chen ZX Zheng JX Chen SR Qu LJ Li JY Xu GY Yang N Low-density lipoprotein receptor-related protein 2 gene is associated with egg-quality traits in dwarf layers Poult Sci 2011 90 12 2718 2722 10.3382/ps.2011-01751 22080009

[B29] Zhang LC Ning ZH Xu GY Hou ZC Yang N Heritabilities and genetic and phenotypic correlations of egg quality traits in brown-egg dwarf layers Poult Sci 2005 84 8 1209 1213 16156204 10.1093/ps/84.8.1209

[B30] Davidson R Mackinnon JG Testing linear and loglinear regressions against Box-Cox alternatives The Canadian Journal of Economics / Revue canadienne d'Economique 1985 18 3 499 517 10.2307/135016

[B31] Wooldridge JM Some alternatives to the Box-Cox regression model Int Econ Rev 1992 33 4 935 955 10.2307/2527151

[B32] Livak KJ Schmittgen TD Analysis of relative gene expression data using real-time quantitative PCR and the 2−ΔΔCT method Methods 2001 25 4 402 408 10.1006/meth.2001.1262 11846609

[B33] Sobel E Lange K Descent graphs in pedigree analysis: applications to haplotyping, location scores, and marker-sharing statistics Am J Hum Genet 1996 58 6 1323 1337 8651310 PMC1915074

[B34] Lu Y Chen SR Liu WB Hou ZC Xu GY Yang N Polymorphisms in Wnt signaling pathway genes are significantly associated with chicken carcass traits Poult Sci 2012 91 6 1299 1307 10.3382/ps.2012-02157 22582286

[B35] Barrett JC Fry B Maller J Daly MJ Haploview: analysis and visualization of LD and haplotype maps Bioinformatics 2005 21 2 263 265 10.1093/bioinformatics/bth457 15297300

[B36] Gabriel SB Schaffner SF Nguyen H Moore JM Roy J Blumenstiel B Higgins J DeFelice M Lochner A Faggart M The structure of haplotype blocks in the human genome Science 2002 296 5576 2225 2229 10.1126/science.1069424 12029063

