# Supplementary figures and images for: The sodium channel gene family is specifically expressed in hen uterus and associated with eggshell quality traits
Source: BMC Genet. 2013 Sep 24;14:90. doi: 10.1186/1471-2156-14-90 (PMC3851161; doi:10.1186/1471-2156-14-90)

Figure A1


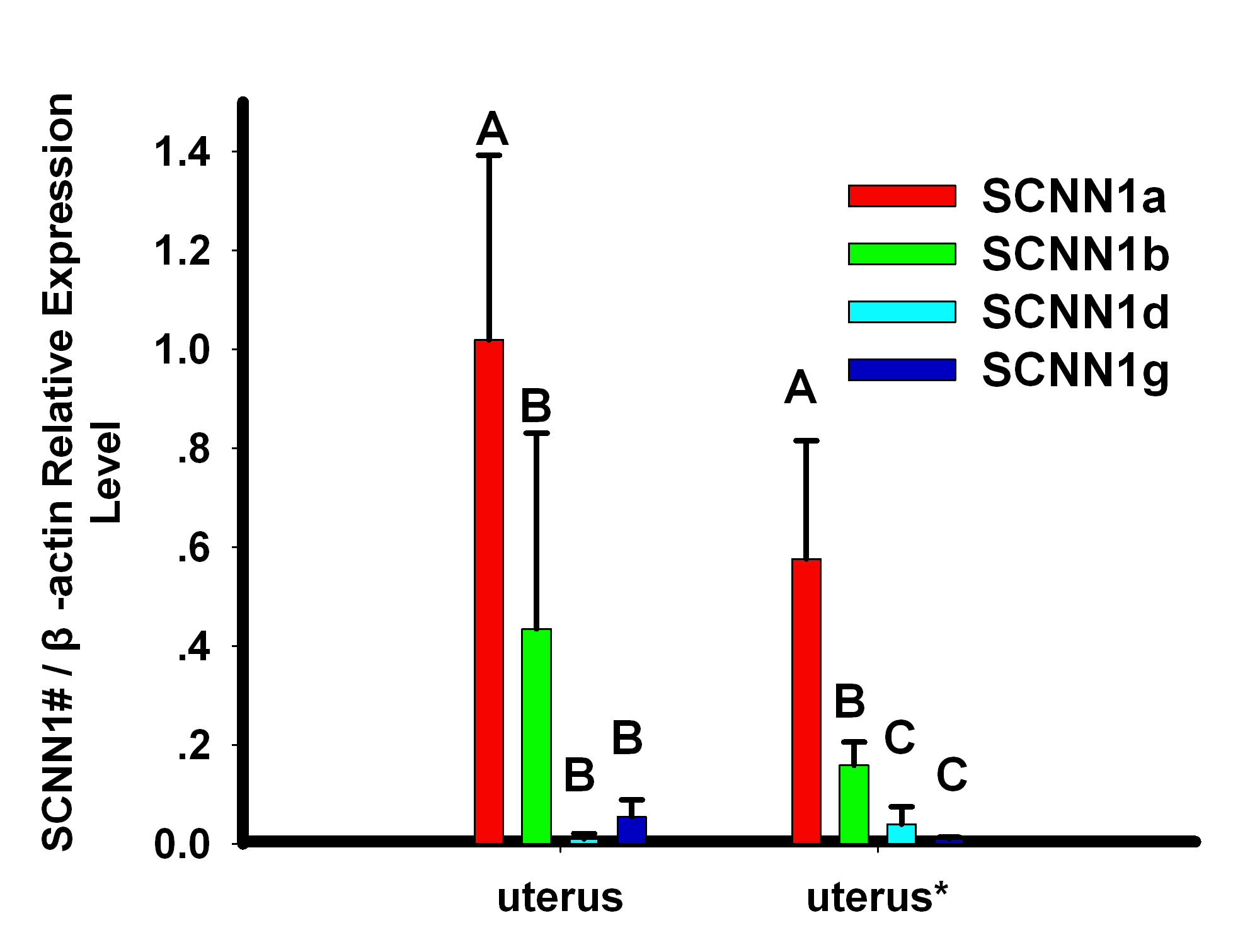

Supplement: Additional file 1: Figure S1 — Comparison of relative expression of SCNN1 gene family members vs β-actin in uterine tissues by qPCR. Gene expression compared between active uterus (during eggshell deposition) and quiescent uterus (no egg present), in tissues collected from four normal 55-week-old White Leghorn layers. The y-axis indicates the relative expression level of SCNN1 family members compared with β-actin. Vertical bars represent the mean ± SD (n = 4). # represent the a, b, d or g. The expression of SCNN1a, SCNN1b and SCNN1g were reduced to some degree in the quiescent uterus, that of SCNN1d increased about 4-fold. The relative expression levels of SCNN1b, SCNN1d and SCNN1g were far less than that of SCNN1a (P<0.05). [file 1471-2156-14-90-S1.doc]

**Figure A2**

**
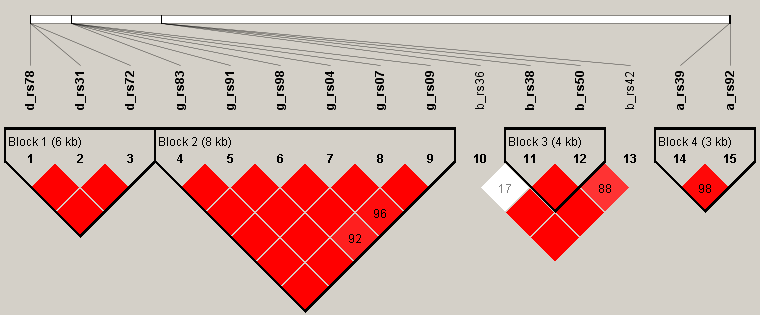
**

Supplement: Additional file 3: Figure S2 — Haploview plot illustrates the linkage disequilibrium of SCNN1 gene family members for the main informative SNPs. The downward-pointing Triangle black box represents LD block. The number in the square represents the value of D’. The darker the square color shows and the larger the D’ value, the higher the level of two sites linkage disequilibrium displays. All SNPs r2 ≥ 0.933 and their minor allele frequencies were > 0.05. In the SNP name, the first letter a, b, d and g are the abbreviation of gene SCNN1a, SCNN1b, SCNN1d and SCNN1g respectively and rs## represents the last two number of the SNP ID. [file 1471-2156-14-90-S3.doc]
