# Supplementary material for: The sodium channel gene family is specifically expressed in hen uterus and associated with eggshell quality traits
Source: BMC Genet. 2013 Sep 24;14:90. doi: 10.1186/1471-2156-14-90 (PMC3851161; doi:10.1186/1471-2156-14-90)
Supplement: Additional file 2: Table S1 — Major haplotypes (frequency >1%) and diplotypes (frequency >0.5%) for SCNN1 family members. [file 1471-2156-14-90-S2.doc]

**Table A1** Major haplotypes (frequency >1%) and diplotypes (frequency >0.5%) for SCNN1 family members.

| Gene | Haplotypes (%) | Diplotypes (%) |
| --- | --- | --- |
| SCNN1a | H1:GC(21.89)  H2:GT(6.66) H3:CC(1.33)  H4:CT(69.97) | H1H1(4.44) H1H2(2.37) H1H4(32.54) H2H4(8.88) H3H2(2.07) H4H4(48.82) |
| SCNN1b | H1:CCCG(8.73)  H2:CCTG(23.82)  H3:CCTA(2.22)  H4:CTCA(30.92)  H5:CTTA(1.78)  H6:TCCA(8.73)  H7:TCTG(1.04)  H8:TTCA(14.05) | H1H2(5.03) H1H4(2.96) H1H6(1.18) H2H2(6.51) H2H4(17.75) H2H6(3.25) H2H8(5.92) H3H4(1.78) H4H4(13.61) H4H8(1.48) H5H1(3.55) H6H6(1.48) H6H8(4.44) H7H4(1.78) H8H8(6.21) |
| SCNN1d | H1:CCCG(8.73)  H2:CCTG(23.82)  H3:CCTA(2.22)  H4:CTCA(30.92)  H5:CTTA(1.78)  H6:TCCA(8.73)  H7:TCTG(1.04)  H8:TTCA(14.05) | H1H2(5.03) H1H4(2.96) H1H6(1.18) H2H2(6.51) H2H4(17.75) H2H6(3.25) H2H8(5.92) H3H4(1.78) H4H4(13.61) H4H8(1.48) H5H1(3.55) H6H6(1.48) H6H8(4.44) H7H4(1.78) H8H8(6.21) |
| SCNN1g | H1:GCCGCC(6.36)  H2:GCCTTT(38.02)  H3:GCTGCC(17.31)  H4:GCTTTT(1.48)  H5:GTCTTT(13.61)  H6:GTTGCC(1.78)  H7:ACCGCC(13.91)  H8:ACCTTT(0.89)  H9:ACTGCC(1.33)  H10:ATCGCC(0.59)  H11:ATCTTT(0.74) | H1H2(1.18) H1H9(2.66) H2H2(14.50) H2H3(15.38) H2H5(12.13) H2H6(3.55) H2H7(11.83) H3H3(5.03) H3H5(2.66) H3H7(5.62) H4H1(2.37) H5H5(3.85)  H5H7(4.14) H7H7(2.37) H8H1(1.78) H10H2(1.18) H11H1(1.48) |
